# Tertiary music students’ hearing health behaviors: ignorance is not bliss

**DOI:** 10.3389/fpsyg.2026.1857791

**Published:** 2026-08-04

**Authors:** Sarah-Ann Soltau, Bridget Rennie-Salonen

**Affiliations:** Department of Music, Stellenbosch University, Stellenbosch, South Africa

**Keywords:** health behavior, hearing conservation, hearing health, music students, musicians, music-induced hearing loss, noise-induced hearing loss

## Abstract

**Introduction:**

Hearing loss is a serious concern among musicians due to the hearing health risks associated with their profession and the high prevalence of music-induced hearing loss (MIHL). Tertiary music students are particularly vulnerable to MIHL due to high sound exposure in educational and recreational settings, yet South African (SA) research on musicians’ hearing health is scarce. This study explored the hearing health behaviors of tertiary music students, investigating how they manage their hearing health, and their attitudes toward and knowledge about their hearing health.

**Methodology:**

Using a qualitative descriptive design and convenience sampling, data were collected through semi-structured interviews with 15 tertiary music students at a university in SA. Interviews were transcribed verbatim and analyzed inductively using Reflexive Thematic Analysis (RTA) resulting in five main themes: Hearing Conservation, Hearing Protection Devices, Music Exposure, Occupational Environment, and Responsibility. The Health Belief Model (HBM) (Janz and Becker, 1984) and the Behavior Change Wheel (BCW) (Michie et al., 2011) together provided a comprehensive framework for the interpretation and discussion of the RTA results.

**Results and discussion:**

Despite acknowledging individual responsibility, students adopted protective measures infrequently, constrained by inadequate knowledge, education, and resources, and a lack of institutional support. Although these tertiary music students recognize MIHL risks, their hearing health behaviors are inadequate. Hearing health promotion strategies, including hearing conservation education embedded in tertiary music education, are recommended for addressing the multi-dimensional contributing factors for optimal hearing health behaviors in the tertiary music setting.

## 1 Introduction

### 1.1 Hearing loss

Hearing loss is a global concern, particularly among young adults (World Health Organization, 2026; Jiang et al., 2023) and is linked to workplace noise exposure (Liu et al., 2025). Occupational noise-induced hearing loss (NIHL) causes approximately 16% of disabling hearing loss worldwide (Chen and Su, 2025). NIHL risk depends on both decibel level and exposure duration (NIOSH: Noise and Hearing Loss, 2026), and standard protocols stipulate a safe limit of 85 dBA for 8 h per day, halved with each 3 dBA increase (World Health Organization, 2022). Temporary noise-induced threshold shifts^1^ can occur (Kurabi et al., 2017), but repeated exposure results in permanent hearing loss (Gopal et al., 2013).

Musicians have specialized hearing requirements yet occupationally are at risk of music-induced hearing loss (MIHL)^2^ (Liu et al., 2025; European Commission: Directorate-General for Education, Youth, Sport and Culture et al., 2023). Orchestral sound levels range from 85 dBA to 110 dBA (McIlvaine et al., 2012), and music teachers’ exposure, including band directors, may exceed weekly safe limits^3^ (Donald and Flagge, 2025; Maffei et al., 2011). Kähäri et al. (2003) recorded peak levels of 148 dBA in a rock/jazz musicians’ context, and in a tertiary student^4^ jazz ensemble, levels exceeded the daily limit (Gopal et al., 2013).

Even slight hearing loss is serious for musicians because accurate hearing is essential to their work (Fitzlaff et al., 2025). Compared to non-musicians, significantly higher rates of hearing loss, including symptoms such as hyperacusis and tinnitus, are reported among musicians, including tertiary music students (McCray et al., 2026:313). A systematic review (Di Stadio et al., 2018) found that 38.6% of professional musicians of diverse genres have MIHL. Firle and Richter (2025) documented MIHL prevalence rates of 5–70% in classical musicians and 20–60% in rock, pop, and jazz musicians, noting methodological heterogeneity and wide-ranging results. Among tertiary music students across instruments and genres, studies demonstrate MIHL prevalence among participants of 45% (Phillips et al., 2010), 63% (Miller et al., 2007), and 86% (Owens et al., 2023). A recent meta-analysis by Kornisch et al. (2024) on hearing loss prevalence in tertiary students, showed significantly higher rates of hearing loss among tertiary music students compared to the general tertiary student population, confirming that student musicians are at higher risk.

Tertiary music students are vulnerable to MIHL due to recreational and educational exposure (Tufts and Skoe, 2018), which increases at university, because music study involves more practice and performance than pre-tertiary years (Phillips et al., 2008), with ensemble settings combining with individual practice (O’Brien et al., 2013). Risk factors include frequency range and intensity level of the music, exposure duration, instrument, genre, repertoire, practice intensity, room size, venue acoustic, ensemble layout, and genetic predisposition (O’Brien et al., 2013; Phillips and Mace, 2008). In ensembles, musicians are exposed to their own and surrounding high-intensity sound (Brusis, 2011); those closest to brass or percussion face higher risk (Schmidt et al., 2014). Individual practice is typically about 3 h daily (Farmer et al., 2014), yet practice-room sound levels often exceed safe limits (Phillips et al., 2010). Certain instrumentalists may be at elevated risk, such as woodwind, brass, and percussion players (Smith et al., 2019). Instrumental playing position is relevant, for example, a violinist’s left ear and a flutist’s right ear are more affected (Schmidt et al., 2011). Pianists’ exposure is mostly linked to their individual practice (Smith et al., 2019). Organists may be vulnerable to MIHL, yet there is limited organ-specific data, with only one known MIHL study which included the organ (Pawlaczyk-Łuszczyńska et al., 2017). Solo and choral singers are also at risk of MIHL, with multiple types of sound exposure including accompanying instruments, loudspeakers, and singers themselves (Hu et al., 2015; Isaac et al., 2017). Recreational contexts cumulatively contribute to young adults’, including music students’, music-related noise exposure (Elmazoska et al., 2024). Concerts, bars, and nightclubs present levels of 100 to 110 dBA (Ramakers et al., 2016), exceeding safe exposure, yet are popular mainly for their loud music (Gilliver et al., 2013). Personal music players also increase MIHL risk through sound levels and exposure duration (Zhao et al., 2012).

These occupational and recreational risk factors for MIHL highlight the importance of hearing conservation for tertiary music students. Effective interventions for musicians integrate education, risk assessment and management, sound control, hearing protection devices (HPD), and audiometric monitoring (Santucci, 2009). Controlling sound at source, through environmental, musical, and organizational strategies, includes managing rehearsal duration, repertoire, and ensemble spacing (Shepheard et al., 2020). Environmental modifications, such as stage layout, increased distance between musicians, use of risers, acoustic treatment with absorptive materials, and acoustic screens can mitigate risk, however, reflective barriers may increase exposure (O’Brien et al., 2012). Musical and organizational factors such as rehearsal scheduling, repertoire, and dynamic intensity can be managed (O’Brien et al., 2012). Therefore, music educators, ensemble directors, and conductors play a central role, highlighting the importance of training educators and institutional leaders in sound control procedures (Chesky, 2010).

The use of HPD can reduce MIHL risk by limiting sound exposure (Pinto et al., 2023), but their effectiveness and acceptability for musicians varies (Greasley et al., 2020). Musicians avoid HPD due to concerns about pitch perception, sound quality, occlusion, tuning, over-attenuation, and cost (MacLeod et al., 2021; Patel, 2008). Consequently, HPD are most effective when integrated with broader sound control strategies (Chesky et al., 2009). HPD use is often reactive rather than preventive, with adoption linked to motivation and training, highlighting the need for education and behavioral change interventions (Olson et al., 2016). Additional behavioral factors associated with musicians’ hearing conservation include modifying both recreational and occupational sound exposure (Olson et al., 2016; Zeigler and Taylor, 2001). Despite musicians MIHL risk, research reports limited uptake and implementation of hearing conservation measures (Greasley et al., 2020; Miller et al., 2007), pointing to the need for investigating musicians’ hearing health behaviors.

### 1.2 Health behaviors

Health behaviors are defined as: “Any activity undertaken by an individual for the purpose of promoting, protecting, maintaining or regaining health” (World Health Organization, 2021:17). Behavior change is thus a fundamental factor in health promotion, including hearing conservation, because “behavior is a critical determinant of health. […] Changes to behavior may either directly benefit health or enable increased control over the determinants of health” (World Health Organization, 2021:17). Consequently, there is a need for comprehensive understanding of the multidimensional determinants of health, which encompass “personal, social, economic, and environmental factors” (World Health Organization, 2021:4). For musicians’ health promotion particularly, a health behavior approach is essential for better understanding the “interplay between myriad factors that influence musicians’ lives” (Norton, 2020:1).

The health behaviors of tertiary music students are of great consequence due to the physical, psychological, and emotional demands of music making (Ginsborg et al., 2009). The high prevalence of performance-related health problems (PRHP) in musicians of all ages, levels, and genres is well documented (Gembris et al., 2018; Kok et al., 2016, 2018; European Commission: Directorate-General for Education, Youth, Sport and Culture et al., 2023), including neuromusculoskeletal, hearing, vocal, and psychological PRHP (Salonen, 2018). Music students face unique demands such as performance pressures, challenging practice schedules, and numerous psychosocial stressors, therefore being vulnerable to PRHP (Ackermann et al., 2024; Ballenberger et al., 2018; Wijsman and Ackermann, 2019). Additionally, students in general report poor health associated with factors such as stress, psychosomatic symptoms, insufficient sleep, and financial worries (Campbell et al., 2022; Mikolajczyk et al., 2008).

South African (SA) students’ health behaviors, including their hearing health, are a relevant concern, because they face context-specific socioeconomic stressors, such as crime, gender-based violence, poverty, inequality, and housing and food insecurity, that negatively affect health and well-being outcomes (Bantjes et al., 2023). SA research on students’ hearing health attitudes and behaviors demonstrates that students lack knowledge on NIHL, that awareness and/or knowledge are insufficient for behavior change, and that behaviorally informed interventions (including hearing conservation programs, education, and policy regulation) are necessary (Khoza-Shangase, 2025; Khoza-Shangase and Mokhethi, 2025a, 2025b; Mahomed and Panday, 2024). Wide-ranging SA hearing health research includes: NIHL in the mining sector (e.g., Grobler et al., 2021; Moroe et al., 2018; Naicker, 2024); childhood hearing health (e.g., Kuschke et al., 2020; Phanguphangu et al., 2025; Roux et al., 2015); and hearing impairments (e.g., Louw et al., 2018; Mtimkulu and Khoza-Shangase, 2023; Ramma and Sebothoma, 2016). However, there is a paucity of research on hearing health in the SA music sector. Although Ntlhakana and Heliopoulos (2020), and Ramma (2021), studied prevalence of MIHL in sound engineers and in Cape Minstrels^5^ respectively, there is no SA research on tertiary music students’ hearing health or hearing health behaviors. Addressing this gap is critical given that literature globally, including SA, documents poor health behaviors among student musicians (Ginsborg et al., 2009; Panebianco-Warrens et al., 2014), and their lacking awareness of risk factors and effective strategies for developing better health behaviors (Araújo et al., 2017; Baadjou et al., 2019; Perkins et al., 2017).

Various barriers hinder tertiary music students’ health behaviors, including cultural influences, perpetuation of ingrained disciplinary norms, and stigma regarding musicians’ health challenges (Evans et al., 2024; Roos et al., 2021). Systemic health promotion approaches in tertiary music settings are recommended, with integration of interdisciplinary health education programs, to support students’ health and well-being effectively (Evans et al., 2024; Lubert et al., 2025). Because health education alone is insufficient for improving health behaviors, incorporating health literacy and self-efficacy conceptual perspectives may be beneficial (Wijsman et al., 2025). Health literacy is an individual’s motivation and capability “to access, understand, appraise, and apply health information” to be able to make well-informed health decisions (Sørensen et al., 2012:3). Musicians’ health literacy is relevant in tertiary music education contexts, where improving musicians’ health literacy can positively impact music students’ and educators’ health behaviors (Guptill et al., 2022; Wijsman et al., 2025). Self-efficacy, associated with health education, health literacy, and behavior change, denotes an individual’s belief in their capability to accomplish a goal, and influences behaviors such as motivation, persistence, and confidence when faced with challenges (Bandura et al., 1997; Zulkosky, 2009). Self-efficacy plays an important role in health literacy competencies, affecting adoption and maintenance of health behaviors (Cudjoe et al., 2020). In tertiary music students, self-efficacy is linked to health behaviors including proper warm-up routines and physical conditioning and is positively correlated with psychological well-being (Panebianco-Warrens et al., 2014; Sun, 2022). Self-efficacy therefore also plays an important role in hearing health behaviors, which are behaviors directed toward conserving hearing health (Coulson et al., 2016).

There is a need to explore tertiary music students’ hearing health behaviors, and how their beliefs and attitudes might influence their motivation for hearing conservation (Gilliver et al., 2013). Health behavior theories are appropriate because they address the psychosocial, cultural, and environmental influences on health-related decision-making (Coulson et al., 2016). This study includes two health behavior change models: the Health Belief Model (HBM) (Janz and Becker, 1984) and the Behavior Change Wheel (BCW) (Michie et al., 2011), which together provide a comprehensive framework for the interpretation and discussion of the results.

#### 1.2.1 Health belief model

The HBM explains health-related behavior by focusing on beliefs and attitudes toward a health issue, viewing behavior as influenced by the value placed on a desired outcome and the belief that a particular action will achieve that outcome (Janz and Becker, 1984). The model comprises six components: perceived susceptibility, perceived severity, perceived benefits, perceived barriers, cues to action, and self-efficacy, acknowledging diverse demographic and psychosocial factors, as well as knowledge of the health condition and its prevention, that influence health beliefs and behaviors (Skinner et al., 2015:78–79). Table 1 outlines the health belief associated with each HBM component.

**Table 1:** HBM components and definitions (Skinner et al., 2015:78).

Component	Health belief
Perceived susceptibility	Beliefs about the likelihood of getting a disease or condition.
Perceived severity	Beliefs about the seriousness of contracting a disease or condition, including physical consequences and social consequences.
Perceived benefits	Beliefs about the positive aspects of adopting a health behavior.
Perceived barriers	Beliefs about obstacles to performing a behavior, and the negative aspects of adopting a health behavior.
Cues to action	Internal or external factors that could trigger the health behavior.
Self-efficacy	Beliefs that one can perform the recommended health behavior.

The HBM suggests that individuals are more likely to engage in preventive action when they perceive a health threat as serious and believe that the benefits of action outweigh the associated costs (Michie et al., 2014). The model is suitable for exploring health behaviors driven by beliefs and attitudes, including engagement with preventive health services and adherence to health advice (Skinner et al., 2015). This is relevant for musicians’ hearing health, as research shows that despite awareness of MIHL, hearing conservation measures are inconsistent (Curk and Cunningham, 2006; Greasley et al., 2020). The HBM has been applied in musicians’ health contexts to examine health behaviors among voice teachers (Schaeffler et al., 2023), and to explore music students’ beliefs about MIHL risk and HPD use (Olson et al., 2016).

#### 1.2.2 Behavior change wheel

**Figure 1:**
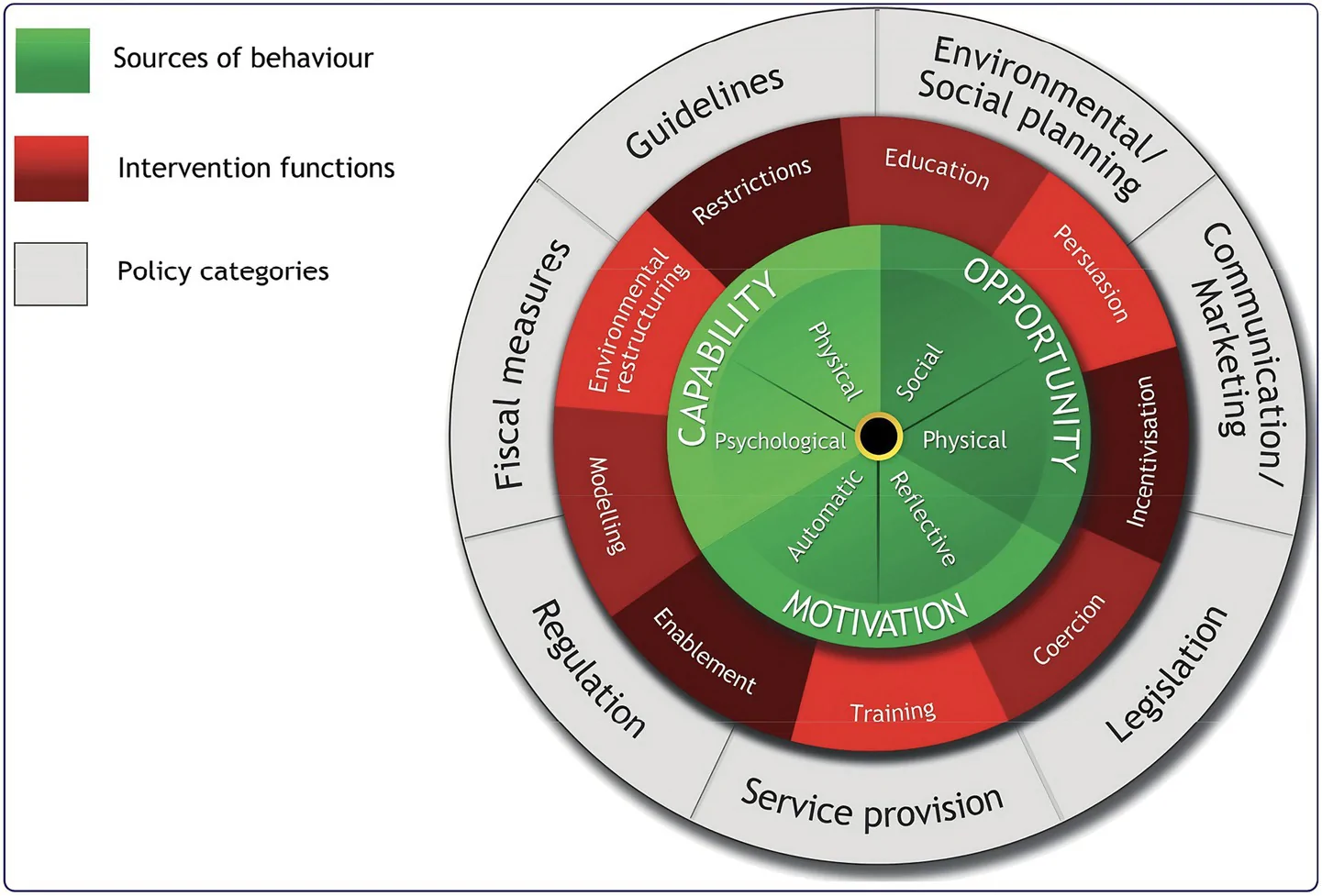
The behavior change wheel (Michie et al., 2011:7).

The BCW explains how behavior is shaped by physical and psychological capability, social and physical opportunity, and motivation (Michie et al., 2011). The COM-B model forms the core of the BCW, demonstrating how behavior change is linked to capability (C), opportunity (O) and motivation (M) (Michie et al., 2011, 2014). To enable behavior change, individuals must be capable of performing it, have the opportunity to do so, and be sufficiently motivated relative to competing behaviors (Barker et al., 2016; Michie et al., 2011). Systemically, behavior change interventions and policies can be characterized by the BCW, comprising the COM-B core, surrounded by intervention functions and policy categories, graphically shown in Figure 1 (Michie et al., 2011).

The BCW is suitable for research on musicians’ hearing health behaviors. Its value has been demonstrated in studies on musicians’ behavior change, including development of interventions for HPD use (Couth et al., 2021); informing interview questions in HPD research (Loughran et al., 2021); addressing cultural factors influencing musicians’ pain experiences (Stanhope and Weinstein, 2020); and investigating musicians’ health promotion interventions (Norton, 2020).

### 1.3 Aims and research questions

The aim of this study is to explore the hearing health behaviors of a sample of SA tertiary music students, guided by the following research questions:

*RQ1*: How do SA tertiary music students manage their hearing health?

*RQ2*: What attitudes do SA tertiary music students have toward their hearing health?

*RQ3*: What knowledge do SA tertiary music students have about their hearing health?

## 2 Methodology

### 2.1 Research design

A qualitative descriptive design was utilized. Guided by constructivism, qualitative researchers emphasize the value of subjective lived experience and assert that meaning and knowledge are socially constructed through human interpretations of, and interactions with, the world around them. A qualitative approach thus requires deep engagement with the subjective realities of participants in their natural setting, allowing researchers to capture and explore the richness, nuance, and complexity of participants’ lived experiences (Denzin and Lincoln, 2005). The researcher is not a detached observer but an active participant in the process of interpreting, understanding, and knowledge-making (Alsop, 2022; Creswell, 2013; Merriam and Tisdell, 2016).

### 2.2 Procedures

Ethical approval was obtained from Stellenbosch University (SU)^6^. Convenience sampling was implemented to invite full-time music students across all undergraduate and postgraduate study programs in the SU Music Department to participate in a 20–30-min semi-structured interview. Potential participants were provided with information and consent forms including voluntary participation, right to withdraw, anonymity^7^, and confidentiality. The final sample comprised 15 participants. See Table 2 for participant demographics.

**Table 2:** Participant demographics.

Name	Year of study: Undergraduate (UG), Postgraduate (PG)	Age	Gender	Instrument(s) or voice	Musical genre
Anna	PG	24	Female	Clarinet, Bass Clarinet, Saxophone	Western art music
Bruno	UG (4th year)	22	Male	Violin	Western art music
Jane	PG	22	Female	Piano, Clarinet	Western art music
Joshua	UG (1st year)	19	Male	Clarinet	Western art music
Lee	PG	25	Male	Flute	Jazz
Lewis	PG	24	Male	Tuba	Western art music
Lucas	UG (3rd year)	24	Male	Voice, Piano, Trumpet	Western art music
Maria	PG	23	Female	Voice	Western art music
Mary	UG (4th year)	22	Female	Violin, Flute, Piccolo	Western art music
Megan	UG (4th year)	21	Female	Voice	Western art music
Noah	UG (1st year)	19	Male	Piano	Western art music
Susan	UG (1st year)	19	Female	Piano, Clarinet	Western art music
Theo	PG	23	Male	Clarinet	Western art music
Tom	PG	24	Male	Trumpet, Conducting	Western art music
Wesley	UG (3rd year)	21	Male	Saxophone, Bassoon	Jazz & Western art music

The interview guide (see Appendix A) was developed from the study aim and research questions, informed by relevant literature on music students’ hearing health, and two pilot interviews. The semi-structured interviews facilitated rich and detailed accounts of participants’ lived experiences, perceptions, and understandings (Braun and Clarke, 2013), allowing for exploration of the participants’ hearing health behaviors, knowledge, and attitudes. The interview guide comprised seven main questions, each supported by three to six prompts. Prompts were used flexibly to probe emerging topics and encourage elaboration during the interviews (Willig, 2013).

Verbatim interview transcriptions were analyzed using Reflexive Thematic Analysis (RTA) (Braun and Clarke, 2019, 2021, 2022). RTA allowed for rigorous qualitative analysis, ensuring the findings were deeply connected to the participants’ perspectives and enabling a rich understanding of the data, valuing the researchers’ subjectivity and interpretation. Analysis followed the six phases outlined by Braun and Clarke (2006): familiarization with the data, initial coding, generating initial themes, reviewing themes, defining and naming themes, and report writing. The first author transcribed the interviews with repeated readings of the transcripts while listening to the recordings for deep immersion in the data; carried out ongoing notetaking of preliminary observations, identifying patterns, and generating an initial set of codes; and refined and sorted codes into a working thematic structure (Braun and Clarke, 2006). Themes were developed inductively and iteratively through reflexive engagement with the data, rather than using a predefined coding framework (Braun and Clarke, 2021).

To ensure “congruent quality practices” (Braun and Clarke, 2025:426) the first and second author together reviewed the coding and interpretation and discussed theme development. As the first author continued with aggregation and refinement of codes, and definitions of final themes with supporting data, the second author was “a sounding board/mirror to enhance reflexivity” maintaining both authors’ deep engagement and reflexive discussions (Braun and Clarke, 2025:426). Detailed notes were kept on interpretive decisions, coding choices, theme development, and peer-debriefing, which were comprehensively documented within the qualitative data analysis software (*Atlas.ti*) throughout the analysis, ensuring methodological transparency, trustworthiness, and rigor (Rose and Johnson, 2020). Yardley (2017:295) similarly describes validity criteria for qualitative research as: “sensitivity to context; commitment and rigor; transparency and coherence; and impact and importance.” Contextual sensitivity was enabled through engagement with relevant literature; deeply probing participants’ lived experiences and “patterns of shared meaning” (Braun and Clarke, 2024:613); careful handling of primary data; and rigorous ethical considerations. Participants reviewed their interview transcripts for accuracy, de-identification, and confidentiality, supporting sensitivity to their data. Consistent with the integral subjectivity of RTA, the authors acknowledged that their own positioning had potential to enhance but also limit their perspectives (Braun and Clarke, 2024). The first author’s positioning as a postgraduate music student familiar with the participants’ context, and the second author’s positioning as a musician experienced in diverse music performance settings, musicians’ health research, and tertiary music education, supported their integrity and sensitivity to context, alongside their ongoing critical self-awareness and collaborative reflexivity.

## 3 Results

**Figure 2:**
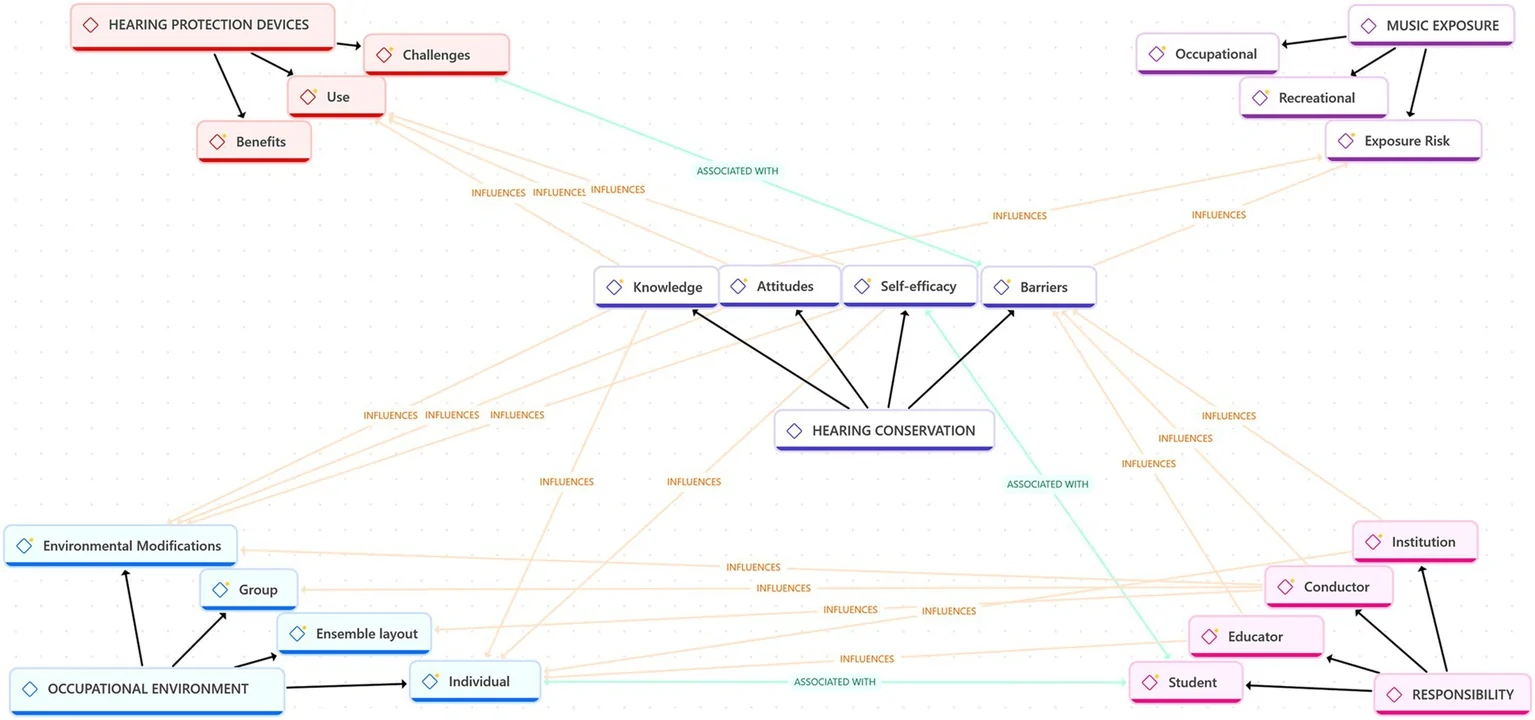
Network illustration of sub-theme interaction.

Data analysis resulted in 18 sub-themes grouped into five main themes (See Table 3). The themes and sub-themes are narratively reported according to the thematic structure. To provide an overview of the relationships identified among sub-themes, a network illustration was developed in *Atlas.ti* (Figure 2), showing associations as well as influences.

**Table 3:** Reflexive thematic analysis overview.

Main themes	Description	Sub-themes
Hearing conservation	Reflects participants’ experiences and understandings of hearing conservation	Knowledge Attitudes Barriers Self-efficacy
Hearing protection devices	Illustrates participants’ experiences and perceptions of HPD	Use Benefits Challenges
Music exposure	Summarizes the participants’ accounts of their occupational and recreational music exposure*	Occupational Recreational Exposure risk
Occupational environment	Captures participants’ perceptions of how their experiences in practice/performance settings shape their understanding of sound exposure	Individual Group Ensemble layout Environmental modifications
Responsibility	Portrays participants’ perceptions of responsibility for hearing conservation	Student Educator Conductor Institution

*The theme ‘Music Exposure’ is descriptive and enumerative, which is not typical of RTA. All the other themes were deeply interpretative and “shared-meaning based themes,” consistent with RTA (Braun and Clarke, 2024:612).

### 3.1 Hearing conservation

#### 3.1.1 Knowledge

Participants demonstrated varying levels of knowledge regarding hearing conservation. While 12 participants^8^ expressed general awareness of risks associated with prolonged exposure to loud sound, 14 reported limited understanding of exposure thresholds, such as Lucas: “*Okay, so I know that you are not supposed to be exposed to a lot of noise for more than, I think it was 2.5 h, I think,*” and Anna: “*I know not to be exposed to like very loud sounds for an extended period of time, especially with earphones. But that’s pretty much what I know.*” Jane reflected: *I know that the amounts of decibels or whatever that we are exposed to is unhealthy [laughs]. And I know that the way I wear my earphones is also not good for my ears.* Similarly, Megan acknowledged an awareness of the relationship between sound level and exposure duration but displayed inadequate knowledge. Nine participants mentioned receiving little to no education on hearing conservation during their musical training. Six participants noted that their only education was one hearing health lecture within the musicians’ health coursework in their final year. Although three of those six participants felt that the lecture was beneficial and comprehensive, Maria described it as brief and introductory, and that it was delivered too late in her studies: *“It would help to have known these things earlier.”*

Five participants reported obtaining information from social media, YouTube, textbooks, and peers. Advice was also sought from a range of musical and health professionals including audiologists and their instrumental/vocal teachers. Despite varying levels of knowledge, 12 participants recognized the potential long-term benefits of hearing conservation education, as Wesley exemplified, “*to benefit me in the long run*.” This was particularly in relation to sustainable careers, like Jane, who expressed a desire to “*preserve the hearing I have because I am a musician*,” and Theo added that education could help “*prevent students from, as musicians, [being] exposed to a lot of loud environments for longer periods of time*,” emphasizing wanting to know how to mitigate potential damage to their hearing. Similarly, Susan said that “*it would help to like have the knowledge so that you can plan ahead in the future*.”

#### 3.1.2 Attitudes

Importance of maintaining their hearing as musicians was acknowledged by 12 participants, describing it as essential for their careers. Lucas emphasized its significance, stating it was “*very, very important*,” while Theo reiterated, “*as a musician, very important.*” However, only five participants made adjustments during practice, such as lowering volume or limiting exposure duration. Maria noted, “*if I have a smaller room then I know I cannot sing at full volume, especially not for a long time*.”

Five participants expressed concern and uncertainty regarding their hearing health. Maria described how inadequate knowledge heightened her “*anxiety*,” while Mary reported persistent worry during prolonged exposure to loud environments, sharing that she had *“been very paranoid.”* In contrast, two participants prioritized musical practice over protecting their hearing, such as Megan: *“It’s more important for me to get a quality practice in than protect my hearing.”* Similarly, Susan continued participating in ensemble activities despite knowing the risks. Feelings of ignorance were captured by Maria, who described an *“ignorance is bliss, but ignorance is not the solution”* approach to hearing health, feeling conflicted, explaining her fear of finding out about irreversible hearing impairment, and cost of audiology assessments, which discouraged taking action:

*I have never been to an audiologist, even though I'm a musician and I should go. Mostly because it's an expensive excursion. Also, it does make me a little bit nervous to think, […] how bad is my hearing or how badly is it damaged, because I do know that it is irreversible. So, I think there's maybe an element of stress that could also be attached to finding out about your hearing*.

Six participants also described disciplinary norms and social pressures that shaped their attitudes. Comments reflected perceptions that hearing loss is normalized or even expected, like Tom: *“inevitably, we are all going to suffer at some stage,”* influencing lack of motivation to adopt protective behaviors. Noah felt that *“it’s sort of in the profession. You do not really have a choice to go around it,”* and Lewis illustrated *“It’s quite risky for brass players. I mean, people say you should get used to it, but [laughs] yeah…”* Despite this, participants acknowledged the long-term importance of hearing conservation, often expressing concern that the consequences of neglect would become apparent later in life.

#### 3.1.3 Barriers

Four participants identified barriers that they perceived as limiting their ability to implement hearing conservation. Financial constraints were common, particularly the cost of audiological assessments and protective equipment. Maria described audiology visits as *“an expensive excursion,”* while others highlighted the challenge of obligatory financial priorities required for their musical training. Environmental and cultural barriers were also evident. Tom described a competitive music environment in which raising concerns about hearing health was perceived by students as detrimental to getting study and performance opportunities, noting fear of being replaced, and pressure to comply with endurance expectations despite knowing the risks:

The music environment, especially in South Africa, is so competitive that many people are actually afraid to complain […] or bring up concerns because you have this kind of in-depth fear of being replaced. And it generally happens where people are replaced simply for being too vocal about problems […] if you complain, if you have any issues, you're just weak and you'll never make it as a professional musician. […] never mind what happens to me in this process in terms of my health or like both physically and mentally, I need to push past the limit to achieve what these people expect.

#### 3.1.4 Self-efficacy

Variable self-efficacy regarding hearing health behaviors was demonstrated by 11 participants. Eight participants described proactive strategies, such as avoiding prolonged earphone use or decreasing volume during individual practice, as Joshua portrayed: “*when practicing in a practice room, I do tend to play softer as I do recognize that the room is smaller.*” However, while four participants acknowledged the importance of protecting their hearing, they admitted to inconsistent or neglected protective behaviors, as Jane reflected: *“I suppose [its] very important, but it’s not really something that I prioritize.”* Linking to a lack of self-efficacy, Maria captured the broader pattern of ignorance among music students by describing hearing conservation as an *“ostrich with its head in the sand”* issue.

### 3.2 Hearing protection devices

#### 3.2.1 Use

Inadequate knowledge regarding HPD types, use, and access was expressed by 14 participants. Bruno acknowledged, *“regarding like professional, hearing [protection] devices, I do not know a lot,”* while Joshua noted that many musicians are unaware of protective options. In contrast, Theo described independently searching for HPD after experiencing discomfort, stating, *“I just Googled music earplugs and then I found different brands.”* However, awareness of alternative or musician-specific HPD was lacking among almost all participants (14). Among those who used HPD (7), only one participant reported using musicians’ HPD, although not custom-fitted, noting that they allowed him to *“hear what the orchestra is doing”* while still feeling protected. Four participants used basic foam/silicone HPD, and two used their earphones (with noise cancelation).

Wide variation regarding when participants used HPD was evident. Eight participants indicated that they did not use HPD at all. Seven participants reported regular use during individual practice or ensemble rehearsals. Usage was often particular to specific instruments or settings, including extended practice sessions and wind band, or when playing louder instruments such as piccolo, trumpet, percussion, but HPD use was selective, erratic, and reluctant, driven by low motivation. Anna expressed that “*I do not wear them when I’m teaching or when I’m conducting*,” and Joshua noted “*I do not wear them during rehearsals*.” Jane described the inconvenience of using HPD in ensemble settings, while Wesley perceived low HPD use as associated with musicians’ resigned attitude to hearing loss as “*just a risk that musicians are willing to take because if it has not negatively impacted us yet, we will not do anything to, you know, prevent it*.”

#### 3.2.2 Benefits

Eight participants identified perceived benefits of HPD use (associated with the use of basic foam/silicone earplugs and not specialized musician-specific HPD), such as reduced sound exposure and less auditory discomfort in high sound level environments. Jane mentioned: “*The advantages are probably that I will just increase my span of having good hearing in my life*,” and Lucas explained that “*It really helps with canceling out some of the noise*.” Anna felt that wearing HPD prevented her headaches, while Megan reported that HPD use mitigated her sound hypersensitivity which she had experienced following noise exposure without using HPD. Bruno described HPD as lowering volume without “*taking the quality of your sound away*,” and Jane described foam earplugs as comfortable for her piano practice sessions. Four participants also recognized hearing protection as important for long-term hearing health, like Joshua stating that HPD support the “*longevity of your… hearing capabilities*.”

#### 3.2.3 Challenges

Challenges of HPD use were highlighted by 14 participants, mostly over-attenuation and the occlusion effect, which were problematic for monitoring intonation and balance within an ensemble. Bruno described difficulty hearing violin overtones during his practice, while Lucas reported challenges detecting pitch accuracy on trumpet: “*I think it’s because it cancels out a lot of what we hear. And as musicians, we kind of depend on what we hear […] maybe I’m slightly sharp, but now I cannot hear it because I have something in my ear*.” Similarly, Anna portrayed: “*I cannot use them in a wind band because then I hear myself in my head and it affects my intonation, it affects how I blend my sound with everyone around me*.” Theo noted that HPD interfered with his ability to hear the conductor or his section, leading to inconsistent use during rehearsals: “*they are not really too nice because I have to take them out sometimes to hear my section to actually work with, um, work with the section or listen to the conductor.*” The occlusion effect was described by Tom (trumpet), who stated that vibrations felt amplified when his ears were uncomfortably “*blocked*” by HPD and “*I feel the sound more in my head*.” Five participants mentioned physical discomfort caused by HPD, including Wesley who expressed that HPD “*tend to hurt my ears*.” Adjustment time was another challenge for three participants, with Lee saying that it took “*a month to 2 months*” to become accustomed to wearing HPD. Financial barriers further limited access to appropriate HPD. Six participants reported purchasing earplugs from pharmacies, reflecting reliance on low-cost foam/silicone options. While three participants expressed interest in high-quality HPD, the cost was widely perceived as prohibitive. As Lee stated, “*the problem of price is a big one*.”

HPD use was also described as uncommon within the tertiary music education environment. Megan described seeing HPD use as “*new*,” while Tom noted that musicians who wore HPD were sometimes mocked, creating a negative social context around their use:

There are people that I've seen in bands or orchestras that I've played in that have worn earplugs in rehearsals, and behind their backs, people often then actually make fun of them […] if something's different, people will find a way to make fun of it. And that creates this negative environment behind the protection, because no one really wants to subject themselves to sort of verbal abuse. So, I think a lot of people make that choice, or even subconsciously, like, …why would I want to do this if I'm then going to become laughingstock?

### 3.3 Music exposure^9^

#### 3.3.1 Occupational

Participants’ occupational music exposure was spread across six musical activity areas: individual instrumental/vocal practice, individual instrumental/vocal lessons, ensemble rehearsals, teaching, conducting, and composing. Individual practice made up the greatest proportion of weekly occupational exposure, with an approximate average of 12 h per week across the 13 participants who spoke about their individual practice. Teaching represented another substantial source of exposure, illustrated by nine participants’ accounts, with their instrumental teaching occupying an average of approximately 8 h per week.

Among the 12 participants who discussed ensemble rehearsal involvement, they experienced an approximate average of 5 h of exposure per week, varying across instrumental specializations. Individual lessons contributed approximately 2 h of exposure per week on average across the 10 participants who mentioned instrumental/vocal lessons. Two participants reported their minimal experiences as student conductors, representing negligible exposure. Similarly, two participants described very slight exposure as composers.

Occupational music exposure varied considerably across participants. Individual practice, teaching, and ensemble rehearsals emerged as the most prominent contributors to occupational exposure, with a combined average total occupational exposure of approximately 23 h per week per participant.

#### 3.3.2 Recreational

Participants’ recreational music exposure was distributed across five main contexts: personal music players, driving, night clubs, church, and Western art music concerts. Listening via personal music players was described as the most prominent source of recreational exposure by nine participants, with an average of approximately 14 h per week. Listening to music while driving represented the second largest source of recreational exposure, reported by eight participants, with an average of approximately 7 h per week. Other recreational contexts, including nightclubs (two participants), church services (three participants), and concert attendance (one participant), were reported as contributing relatively minimal exposure of approximately 1–2 h per week.

Collectively, recreational music exposure averaged approximately 13 h per week among most participants. Personal music player use represented the largest proportion of exposure, while other recreational contexts formed a comparatively small amount of overall exposure.

#### 3.3.3 Exposure risk

All 15 participants perceived occupational music exposure as posing a greater risk to their hearing than recreational exposure. Risk was often attributed to sound produced by other musicians rather than one’s own instrument, although this perception varied by instrument. While nine participants felt external sources posed the greatest risk, others, particularly those playing piccolo, violin, or voice, identified their own instrument as a significant contributor. Bruno stated, “*probably my solo practicing*” (Violin); Mary experienced that “*in wind band, it’s definitely because of my own instrument*” (Flute/piccolo); and Megan attributed that it was “*probably my own practice sessions*” (Voice). Individual practice was also identified as a potential risk. Bruno remarked, “*I can hear less out of my left ear after practicing violin*,” while Mary, who also plays the violin, mentioned, *“my left ear is not so good as my right ear [laughs].”*

Wind band and orchestral settings were frequently described as high-risk environments, particularly due to prolonged exposure to loud sound from surrounding instruments, such as proximity to brass instruments. This was emphasized by Anna: “*Definitely the university’s wind band,*” highlighting the intensity of sound when seated in front of the trombones. Jane characterized “*wind band or orchestra settings*” as involving “*just noise*” for extended periods. Teaching environments were perceived as risky by three participants. This was illustrated by Tom, who expressed concern about his trumpet teaching, stating, “*having multiple students in a very small space*” increases the risk. Wesley shared similar sentiments regarding his saxophone students, noting they “*over-blow a lot*.”

### 3.4 Occupational environment

#### 3.4.1 Individual

Six participants described their individual practice environments as very small, which they perceived as intensifying sound levels and contributing to auditory discomfort during practice. Confined practice rooms were commonly associated with increased loudness and reverberation. Jane remarked, “*there’s no windows in our practice rooms, so sometimes it gets quite loud,*” highlighting her perceptions of spatial and ventilation-related concerns. Maria (voice) acknowledged the existence of larger practice rooms, but due to limited availability, she was often forced to practice in a smaller room. Three participants mentioned adapting their practice behavior in response to their concerns. Lee avoided practicing piccolo in small rooms, stating, “*especially in the practice rooms. It’s so small*,” suggesting an awareness of increased auditory risk associated with limited space. Individual practice was largely undertaken in environments perceived as acoustically unfavorable, with small room size contributing to increased sound intensity and duration.

#### 3.4.2 Group

Group and ensemble rehearsals were frequently described as taking place in environments that amplify sound. Four participants noted that large rehearsal venues, while acoustically responsive, often intensified overall sound levels. Anna described the trombones in such settings as “*exceptionally loud*,” while Tom experienced the main concert hall as “*a brilliant room acoustically*,” but “*too heavy for the size of ensembles that actually frequent it*.”

The two participants involved in teaching or conducting highlighted additional challenges in educational settings, as rehearsals often occurred in spaces that were too large, reverberant, or excessively small, acknowledging how institutional and financial constraints influence rehearsal environments, often resulting in elevated noise exposure during group music-making. Tom portrayed his experiences of ensemble venues being too small for the size of the ensemble, stating:

I think a lot of that does come down to finance as well, where music is not really a priority for many schools. Finances normally seem to get driven more toward sport, […]. So, I think even if there were more venues, they wouldn't be bigger, because the smaller they are, the cheaper it is.

#### 3.4.3 Ensemble layout

Seven participants identified ensemble layout as a contributing factor to auditory risk, particularly their proximity to louder instruments such as brass and percussion. Bruno’s experience was that brass and percussion “*generate the most sound*,” even when positioned at a distance. Susan (clarinet) further illustrated this: “*where the clarinets sit is right in front of like the really loud brass, like right in front of the trombones*.” While two participants described sufficient distancing from intense sound sources, overall ensemble sound levels remained a concern. Brass players such as Lucas, also described challenges related to placement within ensembles, explaining that although he sits behind most instruments, he is positioned directly in front of the percussion, and “*sometimes they bang it really hard*.”

#### 3.4.4 Environmental modifications

There were varying levels of awareness regarding environmental modifications to mitigate sound exposure in musical settings, predominantly with uncertainty or lack of knowledge. Anna stated, “*I know absolutely nothing about that*,” reflecting limited awareness in six participants. Modifications such as risers (for elevated/tiered seating) and acoustic panels (for sound dampening) were commonly experienced yet were largely perceived as visual rather than protective. Jane commented that risers were “*more like a practical thing… so the conductor can see everyone*.” In contrast, eight participants articulated some understanding of how environmental changes could influence sound. Maria discussed the use of sound-absorbing items: “*if there’s carpets and curtains or blinds or anything made of material that can absorb the sound, then bonus*.” Concerns were raised regarding certain modifications in ensemble settings, particularly perspex screens, which were associated with COVID-19 protocols rather than hearing protection. Three participants expressed apprehension that screens could negatively affect sound quality and ensemble cohesion, such as Wesley who noted “*I think it would hinder or impact the sound of the ensemble/orchestra*,” and Joshua: “*trying to implement that without impeding your ability to kind of hear and listen out for your fellow players, …might be a big challenge*.”

### 3.5 Responsibility

#### 3.5.1 Student

All 15 participants perceived themselves as responsible for managing their own hearing health, expressed explicitly and often with confidence. Lucas stated, “*I am most definitely responsible for managing my hearing health*,” while Megan emphasized the importance of self-advocacy: “*if you do not speak up, I do not think it’s necessarily the person in charge’s responsibility*.” However, perceived responsibility translated into adaptive behaviors during practice in only six participants. Strategies included modifying playing volume in small spaces and using practice mutes to reduce sound exposure. Their accounts indicate that they not only accepted their responsibility, but also actively implemented personal strategies to attempt to mitigate risk.

#### 3.5.2 Educator

Participants expressed mixed views regarding educators’ responsibility for promoting hearing conservation. Six students, including Anna, perceived a lack of awareness or concern among instrumental/vocal teachers, suggesting that increased awareness was needed, but worrying that raising the issue might be met dismissively: “*they’d probably roll their eyes at me*.” Referring to his assumptions about his teacher, Lee mentioned that he “*did not expect him to, like, care about health*” because his teacher was “*so old*.” However, four participants described supportive experiences, particularly with educators who actively addressed sound exposure in their teaching. Lewis’s teacher recommended regular hearing tests and demonstrated extensive knowledge of hearing conservation. Bruno said that his teacher “*suggested […] not practicing for hours and hours on end,*” and Maria expressed that “*one of the things she mentioned when she spoke to me about practicing, was I need to make sure I’m modifying my instrument according to the size of the space.*”

#### 3.5.3 Conductor

Seven participants experienced conductors playing a limited role in managing hearing conservation within ensemble settings, recalling that conductors rarely addressed volume or encouraged safer listening practices. Jane summarized this perception succinctly, stating, “*they just let it happen*.” In contrast, the two participants who identified as student conductors described a more intentional approach, as they reported carrying out sound checks, adjusting dynamics according to venue size, and explicitly addressing ensemble volume.

#### 3.5.4 Institution

According to four participants, hearing health was rarely acknowledged in institutional contexts, particularly within large ensemble settings such as wind band and orchestra rehearsals. The absence of warnings, guidance, or formal consideration of hearing health contributed to the students’ perceptions that institutional responsibility was minimal. They expressed a desire for greater institutional involvement in hearing conservation, and their need for better understanding of the institutional responsibility. There was a perceived expectation that the institution should play an enabling and educational role, as stated by Jane “*it would be nice if that was considered*.” Joshua expressed a desire for greater institutional accountability, stating, “*I would be satisfied if they were to also take my hearing health into account*,” in reference also to music event organizers. Noah emphasized the importance of the tertiary music institution providing education and access to protective strategies: “*to enable… the protection of the ears; you know, they need to educate people so that they know how to do so*.

## 4 Discussion

**Figure 3:**
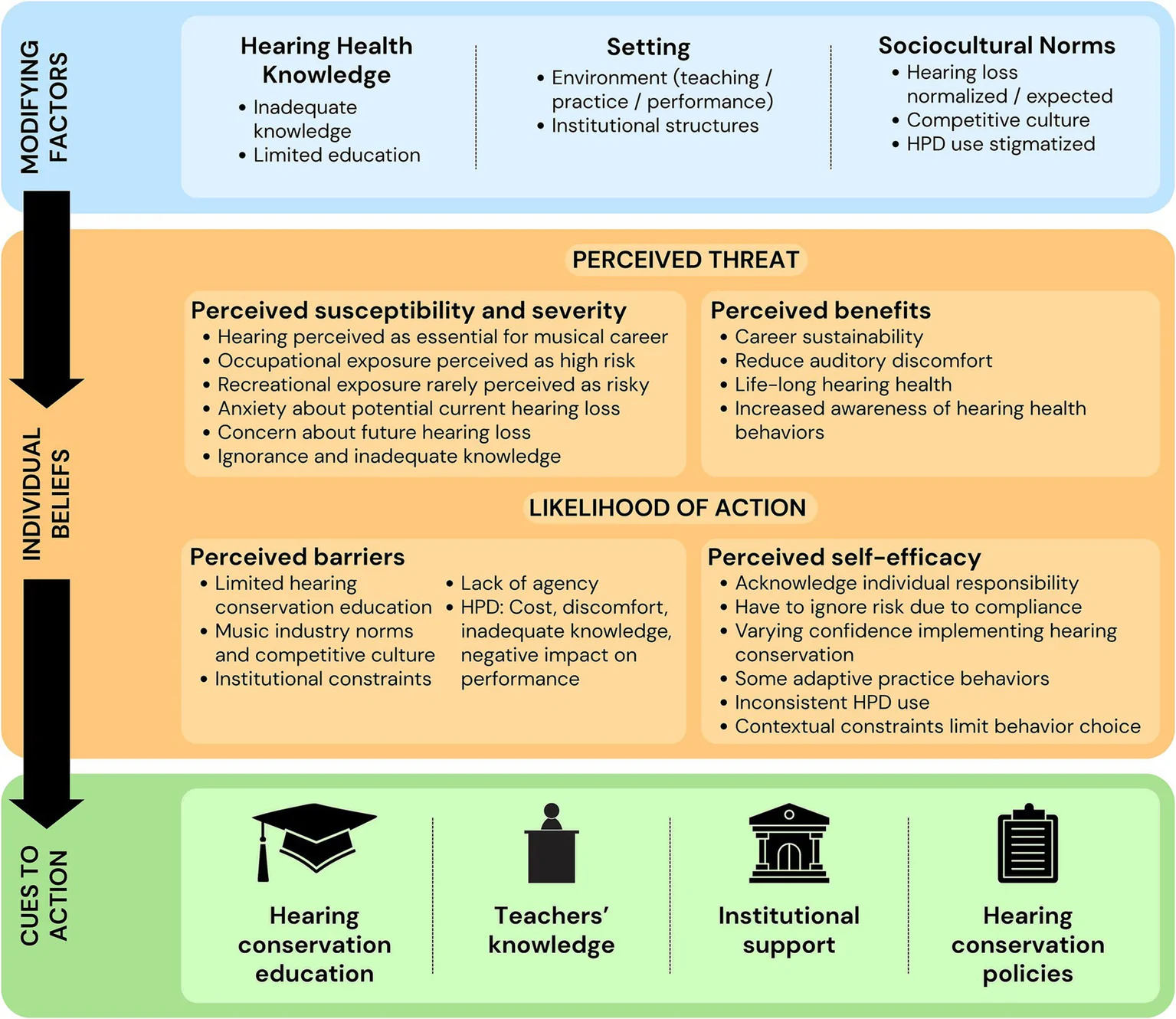
An overview of the results understood alongside the HBM components.

This study aimed to explore the hearing health behaviors of a sample of tertiary music students, investigating how they manage their hearing health, what attitudes they have toward their hearing health, and what knowledge they have about their hearing health. Each of the three research questions is addressed and discussed below. The HBM (Janz and Becker, 1984) and BCW (Michie et al., 2011) health behavior theoretical frameworks are used as an informative lens to critically discuss and interpret the results in relation to the research questions, integrating relevant literature (Figure 3).

### 4.1 Managing hearing health (RQ1)

Findings indicated that these tertiary music students self-reported substantial occupational and recreational sound exposure, placing them at potential risk of MIHL. Exposure came mainly from individual practice, ensemble rehearsals, teaching, and use of personal music players. Although sound levels and exposure durations were not quantitatively measured, the participants’ self-reported approximate duration and contexts of exposure, when considered alongside documented sound levels in similar settings, strongly suggest cumulative risk. This is consistent with results from prior studies (Firle and Richter, 2025; Phillips and Mace, 2008; Phillips et al., 2010; Størmer et al., 2015). Research confirms that occupational MIHL affects music students who practice for extended periods and participate in multiple ensembles (McIlvaine et al., 2012; Tufts and Skoe, 2018; Washnik et al., 2016). Teaching also emerged as a high exposure context, aligning with studies on music educators’ sound exposure (Behar et al., 2004; Maffei et al., 2011). Our study results emphasize that participants’ exposure involved several occupational contexts and recreational activities which together intensified exposure. Personal music players and attendance at clubs and concerts added to weekly sound load, consistent with prior work identifying these as high-risk behaviors among young adults (Gilliver et al., 2013; Zhao et al., 2012).

Importantly, although all 15 participants described their recreational sound exposure, no participants perceived it as a risk to their hearing health. Because participants tended to view occupational and recreational exposures separately, none understood cumulative exposure, suggesting that students’ awareness of potential risk was often disconnected from their everyday life. This lacking competence in cumulative risk appraisal may reduce the uptake of preventive hearing health behaviors, despite general awareness of risk. This shows the students’ misconceptions in terms of their perceived susceptibility, a key construct within the HBM (Janz and Becker, 1984).

Perceptions of exposure risk were shaped by environmental factors such as small rooms, reverberation, ensemble size/layout, and instrument type. This aligns with previous research which reports that risk differs from player to player due to multiple extrinsic factors (Zbysińska and Lachowska, 2020). Similarly, according to the HBM (Janz and Becker, 1984), various extrinsic *modifying factors* will influence the likelihood of an individual’s health behaviors. Musicians frequently rehearse in multi-purpose venues with diverse acoustics, such as school halls and classrooms (Laitinen and Poulsen, 2008). Excessive reverberation in unsuitable venues can elevate perceived and measured sound levels (Gade, 2012; Smith et al., 2019). Participants also illustrated multiple challenging experiences of trying to manage their sound exposure related to ensemble layout. Research similarly highlights layout risk mitigation strategies, such as placing brass instruments on risers (O’Brien et al., 2012). Participants’ perceived exposure levels due to instrument type was evident, with violin and flute/piccolo players experiencing that their hearing was considerably worse on the side that they hold their instrument, consistent with reported unilateral hearing issues in these groups (Schmidt et al., 2011; Zinn-Kirchner et al., 2023).

There were widely varying levels of access, acceptance, and confidence in HPD use, ranging from consistent use in certain settings, to irregular use or not at all. None of the participants reported access to or experience with audiologist prescribed custom-molded HPD, preventing any discussion of participants’ perceptions of musician-specific HPD use. Approximately one third of the participants relied on basic foam/silicone earplugs, suggesting limited use of musician-specific HPD due to resource constraints in the SA socioeconomic context. The *perceived benefits* of HPD were based on assumptions about the effectiveness of basic foam/silicone earplugs. The HBM conceptualizes *perceived benefits* as a key determinant of behavior change (Janz and Becker, 1984), yet these students’ lack of experience with high-quality HPD meant that *perceived benefits* were minimal and meaningful hearing health behavior change did not occur. Although these basic options are affordable and accessible, they are inadequate for musicians. There is a clear need for education on effective and correct HPD use.

Barriers to HPD use were related to financial, cultural (musician and institutional), and practical challenges, demonstrating how hearing conservation was controlled by systemic norms as much as by individual choice. Financial constraints were prominent, as these SA students’ sociodemographic factors impacted their access to specialized musicians’ HPD and constrained their broader preventive hearing health behaviors. Participants described competitive disciplinary norms and fear of disclosure regarding health concerns, aligning with research highlighting systemic barriers within music education environments (e.g., Lubert et al., 2025; Perkins et al., 2017). The HBM (Janz and Becker, 1984) provides a framework to enhance understanding of how the stigmatization of HPD use and expectations of musician culture function as *modifying factors* for *individual beliefs* about hearing health behaviors. Participants expressed concerns about reduced musical control due to pitch distortion, volume alteration, and the occlusion effect, similarly demonstrated in the literature (e.g., MacLeod et al., 2021; Patel, 2008). Adjustment to HPD use was also described as challenging, supporting evidence that adaptation periods are necessary for sustained use (Olson et al., 2016). *Perceived barriers* to the uptake of HPD are framed clearly in the HBM (Janz and Becker, 1984) within *individual beliefs* and include cost, discomfort, inadequate knowledge, and negative impact on performance.

Overall, these participants demonstrated awareness of the risk of MIHL but faced systemic constraints and motivational barriers influenced by cultural norms and perceived disadvantages of using HPD, therefore limiting the translation of awareness into action. Prior research demonstrates this gap between musicians’ awareness and their protective hearing health behaviors (e.g., Greasley et al., 2020). Within the BCW framework, *capability*, *opportunity*, and *motivation* as *sources of behavior* must align for protective behavior to become sustainable (Michie et al., 2011, 2014). Environmental modifications were also poorly understood, and participants had inadequate knowledge, highlighting that education on environmental controls is needed. Due to inadequate knowledge of hearing conservation, preventive behaviors were inconsistent, suggesting students’ variability in self-efficacy, and limited ability to translate awareness into action. The HBM (Janz and Becker, 1984) perspective illustrates how their inadequate knowledge and limited education function as *modifying factors* and clarifies the need for hearing conservation education as part of *cues to action*.

Hearing health management among these students is insufficient. Although they are aware of the need for hearing conservation, their behaviors are shaped by inadequate knowledge, financial and resource constraints, musician culture, performance demands, and lack of institutional support. Hearing health behaviors are usually reactive, fragmented, and likely to be ineffective, as students are not equipped with the necessary competencies.

### 4.2 Attitudes toward hearing health (RQ2)

Participants recognized the importance of hearing conservation and were motivated to protect their hearing, however, this was not sufficient to overcome institutional norms and other barriers that limited action. Viewed through the BCW (Michie et al., 2011), *motivation* was present among students, but *capability* and *opportunity* for preventive action were often lacking. Attitudes toward hearing conservation varied widely: some students were proactive, while others were ignorant of the risks to their hearing, consistent with previous studies showing music students’ inconsistent uptake of protective behaviors (e.g., Olson et al., 2016). Engagement in preventive behaviors is influenced by *perceived threat (susceptibility and severity)*, as described in the HBM (Janz and Becker, 1984). Some participants showed limited awareness of the potential risk of MIHL, suggesting that the *perceived threat* component may not be sufficiently significant to prompt protective action.

Although participants acknowledged their own responsibility for their hearing health and their efforts were well-intentioned, they were mostly inconsistent and misguided and did not feel sufficiently supported. Students described some proactive approaches, such as adjusting volume according to venue size and using practice mutes, reflecting moderate *capability* and *motivation*, but limited *opportunity,* the three components within the BCW (Michie et al., 2011) that require alignment for sustained behavior change.

Emotional responses to hearing conservation reflected a tension between musical training demands and attempts to protect hearing. Although participants acknowledged the long-term importance of hearing for career sustainability and recognized the serious consequences of neglecting hearing health, performance prioritization often took precedence over preventive action. Participants experienced cognitive dissonance, wanting to act but feeling conflicted by fear of disclosure, inadequate knowledge, and systemic barriers. Ignorance due to inadequate knowledge as well as having to ignore risk due to compliance, fueled frustrating feelings of resignation, disempowerment, and lack of agency: Ignorance in this situation was not bliss. The *modifying factors* component of the HBM (Janz and Becker, 1984), specifically disciplinary norms, influenced students’ attitudes, emphasizing that musician culture change is needed alongside individual behavior change (Stanhope and Weinstein, 2020).

Educators were recognized as important influences, and participants reported both supportive and dismissive attitudes from their instrumental/vocal teachers, suggesting that hearing conservation is not yet accepted within the music environment. Within the HBM (Janz and Becker, 1984), educators act as *cues to action*. Additionally, within the BCW (Michie et al., 2011), through *modeling,* educators can nurture *capability*, *opportunity*, and *motivation* for protective health behaviors. Training educators in noise reduction strategies for them to be able to teach and model healthy behaviors is critical (Ginsborg et al., 2009; Hayes, 2013), particularly because educators themselves are exposed to high sound levels and need to be active participants in hearing conservation rather than passive observers (Behar et al., 2004; Maffei et al., 2011).

Participants highlighted the conductor’s influence on ensemble sound levels and expressed concern that they did not foster safer listening environments. Conductors act as risk regulators, controlling dynamics and overall sound exposure (Chesky, 2010), so their role is central to whether hearing conservation is normalized in rehearsals. Controlling sound at the source, through dynamics, sound balance, and repertoire planning remains one of the most effective ways to reduce risk within the ensemble (Shepheard et al., 2020). Conductors’ own exposure to high sound levels reinforces their need for hearing health knowledge and positive attitudes toward hearing conservation (Hayes, 2013; Owens, 2004).

Institutions were identified as key in supporting hearing conservation, illustrated as a *cue to action* in the HBM (Janz and Becker, 1984), especially in the SA context, where institutional budgets, venue quality, healthcare disparities, and access to resources are common barriers. These *perceived barriers* are also a component of the HBM (Janz and Becker, 1984). Institutions can provide practical support in the form of appropriate venues, acoustically safer rehearsal spaces, and access to HPD. Legal duty of care and occupational health regulations require risk assessment and surveillance where exposure may be hazardous (Barlow, 2010; Chesky, 2010; Shepheard et al., 2020). However, because music students are not classified as employees, they may not be protected by occupational noise regulations (Kovalski et al., 2025). Institutional support is therefore important, as institutions shape the *opportunity* component of the BCW (Michie et al., 2011) by providing *training*, resources, and the physical environment in which students rehearse and perform. Integrating health promotion programs—*cues to action* from the HBM (Janz and Becker, 1984)—into music curricula strengthens students’ capacity to manage occupational risks, including MIHL (Evans et al., 2024; Guptill et al., 2022).

Hearing health behaviors were not only about individual attitudes and choices. Motivated and concerned about hearing conservation, participants’ attitudes were linked to inadequate knowledge, and varying capability, and they had to contend with performance priorities, educators, conductors, and the institution. Students were expected to manage risk individually, while educators, conductors, and institutions did not consistently make hearing health a visible part of training, rehearsal culture, and the learning environment. Hearing conservation depends on systemic change in which proactive hearing health promotion is embedded as part of musicianship in the tertiary music environment.

### 4.3 Knowledge about hearing health (RQ3)

Participants had inadequate knowledge, aligning with previous research indicating widespread concern about musicians’ limited knowledge of hearing conservation (Greasley et al., 2020; Miller et al., 2007). Because knowledge functions as a *modifying factor* for health behaviors in the HBM (Janz and Becker, 1984), participants’ inadequate knowledge impacted *perceived threat* and *likelihood of action*. They obtained advice from educators, social networks, and online sources, primarily consulting their teachers for guidance, but the quality and usefulness of this input varied. Importantly, participants had not received sufficient hearing conservation education. Those who applied what they knew demonstrated low self-efficacy, which did not translate into sustained prevention. Within the HBM (Janz and Becker, 1984), *perceived self-efficacy* represents confidence in performing the recommended behavior, a driver of health behavior change, which was lacking in these participants.

Health behavior research demonstrates that knowledge acquisition alone does not necessarily link to health behavior change or improved health outcomes (Evans et al., 2024), and that applying a health literacy framework— “the knowledge, motivation and competencies to access, understand, appraise, and apply health information” (Sørensen et al., 2012:3)—is beneficial for investigating student musicians’ health behaviors (Wijsman et al., 2025). These students were able to access information and begin to understand hearing health principles, but their ability to critically appraise and apply musician-specific strategies was deficient, therefore limiting their *self-efficacy* and *capability* to implement effective hearing conservation. Improved hearing health literacy is therefore needed to support effective hearing health behaviors. Viewed through the BCW (Michie et al., 2011), these students’ *motivation* was moderate, but their *capability* and *opportunity* were limited by the lack of training and resources, while *intervention functions* such as *education*, *enablement*, and *modeling* were largely absent. Participants themselves acknowledged the fundamental need for comprehensive hearing conservation education—the *capability* component in the BCW (Michie et al., 2011) and a *cue to action* from the HBM (Janz and Becker, 1984)—at the start of their tertiary studies to equip them with the appropriate knowledge.

These findings emphasize the imperative for early, mandatory, musician-specific hearing conservation education, integrated into tertiary music curricula, to drive sustained protective hearing health behaviors. Effective hearing conservation implementation in tertiary music settings needs to move beyond raising awareness and toward applied hearing conservation education, tailored to specific genres, instruments, and environments.

### 4.4 Limitations

The research was limited by the small sample size in a single institution which prevents the generalizability of the results; however, they may be transferable to similar contexts. The study captures only students’ self-reported perceptions of loudness and exposure. Literature confirms that subjective and dosimetric estimates do not always align due to inaccuracy of self-reported perceptions. Therefore, self-report should not be treated as a substitute for measured exposure (Portnuff et al., 2013). No prior exposure, audiometric or other objective data about participants’ hearing status was measured, so symptoms such as tinnitus or hyperacusis, and prior audiograms, could not be incorporated in the data. Potential biases of convenience sampling may reduce the robustness of the conclusions. A small sample size allowed for rich and detailed insights, however, diversity of experiences within a SA tertiary music setting may not have been captured, as the majority of participants were primarily studying Western art music, and most of the instrumentalists were woodwind/brass players. Given that MIHL risk profiles differ across genres and instruments, these sample characteristics should be acknowledged as a limitation.

### 4.5 Future directions

Further research with larger, randomized, and diverse samples is necessary to investigate tertiary music students’ hearing health and hearing health behaviors across musical genres, institutions, cultural contexts, socioeconomic backgrounds, and underrepresented groups, including research comparing professionals and students. Longitudinal studies would enable tracking of hearing function and risk factors over time, such as multi-year prospective cohort studies with baseline assessments and regular follow-ups. Potential topics include exposure characteristics in different ensemble settings examining personal noise dosimetry during rehearsals and performances; music students’ personal listening device use; recreational MIHL among music students; as well as HPD effectiveness, usability, and evaluation of different HPD designs alongside technology-enhanced hearing protection and monitoring solutions.

Mixed-methods implementation science research is needed for effective translational research impact, such as understanding how hearing health education can be realistically integrated into music curricula despite prevalent institutional, resource, and healthcare constraints; broader conceptual framing of musicians as professional ear users; as well as developing context-specific adaptations. Qualitative and quantitative approaches can be combined, for example utilizing semi-structured interviews, focus groups, or other participatory methods with all stakeholders in the tertiary music setting, alongside validated quantitative tools, objective outcome measures, dosimetry, and clinical assessments. Such research would aid in identifying specific population needs, such as in SA, understanding barriers and facilitators to implementation, as well as assessing effectiveness of hearing conservation interventions.

## 5 Conclusion

These tertiary music students acknowledged their responsibility for their hearing health and were motivated, demonstrating some proactive measures, but their hearing health behaviors were constrained by barriers such as ignorance, inadequate knowledge, limited resources, musician culture, performance prioritization, and insufficient institutional support. Awareness of the risk of MIHL did not translate into consistent protective or sound-control strategies, as students were left to manage risk individually in a system where hearing conservation was not embedded, and potential for MIHL was normalized and accepted.

The implementation of hearing conservation interventions—including education integrated into core curriculum from the start of tertiary music training and tailored to context—is needed to support the hearing health behaviors of tertiary music students. Enabling equitable access to hearing healthcare (including regular audiological assessments) and affordable musician-specific HPD is needed for music students in socioeconomically challenging environments such as in SA. The design and implementation of programs would benefit from multidisciplinary collaboration to better understand the multi-dimensional determinants influencing musicians’ hearing conservation. Systemic enablers for hearing health promotion in tertiary music settings would include leader, educator, and conductor training in hearing conservation; provision of acoustically safe teaching and rehearsal spaces; as well as establishing guidelines for educational and behavioral interventions. Underscored by a health promotion approach, these initiatives would support both optimal health and optimal performance among tertiary music students.

## Data Availability

The datasets presented in this article are not readily available because the datasets generated for this study are not available due to ethical and confidentiality restrictions. Participants did not consent to the sharing of raw data with third parties, and access was limited to the researcher and supervisor. All data were de-identified, and only anonymized excerpts are presented in the article. In accordance with the approved protocol, raw data are not publicly available. Requests to access the datasets should be directed to sasoltau99@gmail.com.

## Footnotes

1 Post-exposure threshold recovery may conceal underlying persistent neural damage. Cochlear neurodegeneration after “temporary” noise-induced threshold shifts is a hidden consequence of noise exposure, referred to as ‘hidden hearing loss’ (Kujawa and Liberman, 2009).

2 MIHL refers specifically to hearing loss caused by overexposure to high-intensity music and is considered a subtype of NIHL. The main distinction between NIHL and MIHL is the exposure source (Reynolds and Bielefeld, 2023).

3 Further research is recommended to determine whether the weekly safe limit established for NIHL can be directly applied to MIHL (Strasser et al., 2003).

4 The term tertiary music students will be used throughout to refer to students enrolled in post-secondary (higher education) music study programs (e.g., universities, conservatoires, or colleges).

5 Cape Minstrels are community-based wind ensembles who come together as part of a historic community tradition to create and perform music during the annual Cape Town Minstrel Carnival (Ramma, 2021).

6 SU Faculty of Arts and Social Sciences Research Ethics Committee: Social, Behavioral and Education Research (SU project ID: 29473).

7 Participants’ names were replaced with pseudonyms.

8 The extent of particular experiences and perceptions within the group is indicated by providing specific participant numbers in order to show how widely the sub-themes occurred. Although this is not typical in RTA (Braun and Clarke, 2024), it provided clarity for reporting the results and enhanced understanding for addressing the research questions.

9 Note that the approximate exposure ranges originate from semi-structured interviews with participants and are therefore self-reported time estimates only.

## Appendix A: Semi-structured interview guide

**Table:**

Main questions		Prompts
		Generic: *Tell me a bit more about that? What do you mean by that? How do you feel about that?*
1	Could you tell me about your musical background?	*What instrument(s) do you play/which voice type are you?* *How long have you been playing/singing for?* *What year/program are you in?* *What style(s) of music is your primary focus?*
2	What singing/playing-related activities are you involved in?	*How many hours a week do you spend playing/singing/conducting?* *How much of that is spent rehearsing in/practice room/teaching/performing/alone/group?* *Which of your musical activities do you perceive as the greatest risk to your hearing?*
3	What recreational music are you exposed to?	*How many hours are you exposed to a week?* *Do you use earphones/headphones when listening to music?* *How often do you attend clubs or other venues that play loud music?*
4	How important do you think it is for you to look after your hearing?	*What do you know about hearing health?* *Who do you think is responsible for managing your hearing health?* *What is your source(s) of information/education regarding hearing health?* *When did you receive education on MIHL in your music career?* *How do you think having an understanding about hearing health benefits you as a music student?* *Who do you consult for the care of your hearing?*
5	What do you know about hearing protection devices?	*Do you make use of any hearing protection devices (HPD)? What type?* *When do you wear HPD? (Do you wear HPD outside of rehearsals, practice, etc.?)* *How long did it take for you to get used to wearing HPD?* *What are your perceived advantages/disadvantages of HPD?* *What are your reasons for wearing/not wearing HPD?* *Why do you think the uptake of HPD is low, even with awareness of the risk of MIHL?*
6	What do you know about environmental modifications for hearing protection?	*What environmental modifications do you make in practice/rehearsal/performance?* *What environmental modifications do your ensembles make?* *What environmental modifications do your lecturers make?*
7	What do you know about musical modifications for hearing protection?	*What musical modifications do you make in practice/rehearsal/performance?* *What musical modifications do your ensembles make?* *What musical modifications do your lecturers make?*
